# Low-Cost Scalable Radiative Cooling Membrane via Spray Fabrication for Sustainable Thermal Management

**DOI:** 10.3390/ma18184385

**Published:** 2025-09-19

**Authors:** Liang Lv, Jiaqi Hu, Ruichen Song, Xusheng Xia, Zhilin Xia, Siyuan Yu

**Affiliations:** 1 School of Astronautics, Harbin Institute of Technology, Harbin 150001, China; lianglv_hit@163.com; 2 State Key Laboratory of Advanced Glass Materials, Wuhan 430070, China; hujiaqi@whut.edu.cn (J.H.); 285550@whut.edu.cn (R.S.); xiazhilin@whut.edu.cn (Z.X.); 3 School of Material Science and Engineering, Wuhan University of Technology, Wuhan 430070, China; 4 School of Instrument Science and Engineering, Harbin Institute of Technology, Harbin 150001, China

**Keywords:** radiative cooling, passive thermal management, spray-coated films, infrared transparent materials, zirconia nanoparticles

## Abstract

Radiative cooling presents a promising passive cooling strategy, though its widespread adoption is often constrained by elevated costs and manufacturing complexities. This study introduces a cost-effective, scalable fabrication method for a composite membrane utilizing a spraying technique, and it was fabricated by spraying a mixture of modified nano-zirconia and ethylene-octene copolymer (POE), dissolved in petroleum ether, onto a polyethylene (PE) bubble film substrate. This composite membrane demonstrates a hydrophobic property, with a water contact angle of 100.6°. A cooling structure was formed by covering the composite membrane onto a polytetrafluoroethylene (PTFE) plate which served as an emitter, and the cooling power of this structure reaches 66.2 ± 4.3 W/m^2^. Field tests reveal a temperature reduction of 3 ± 0.3 °C at noon and an average cooling effect of 4.7 ± 0.3 °C throughout the day, relative to ambient temperatures. This work advances the development of cost-effective, scalable radiative cooling technologies, holding promise for applications in building cooling and energy efficiency.

## 1. Introduction

As global climate change progresses, the annual demand for cooling solutions is experiencing exponential growth. Conventional air conditioning systems are characterized by their substantial energy consumption; the surging electricity demand consequently results in significant greenhouse gas emissions, further intensifying the global warming crisis [1,2]. Hence, there is an urgent need to develop energy-efficient and high-performance cooling technologies.

Radiation cooling, as an efficient and zero-energy technology, has garnered significant attention. To achieve daytime cooling, high solar reflectance in the 0.3–2.5 μm range and high emissivity in the 8–13 μm atmospheric window are essential for reflecting solar radiation and emitting heat to the sky [3,4,5,6,7]. This passive cooling process requires no external energy, offering a promising zero-energy thermal management solution to minimize fossil fuel consumption and mitigate environmental pollution.

Researchers have extensively explored various approaches to enhance the efficiency of radiation cooling technology. Typical cooling materials include inorganic pigments such as Al_2_O_3_, TiO_2_, BaSO_4_, CaCO_3_, and ZrO_2_ dominate radiative cooling strategies due to their excellent solar reflectance. Organic polymer-based systems including porous films or PE aerogel. Organic–inorganic composites blend mechanical robustness with spectrally optimized performance. Polymeric films functionalized with reflective fillers, such as Al_2_O_3_ in polymers, provide scalable production routes. A summary of representative material types, their optical characteristics, and fabrication methods is provided in Table 1.

By leveraging the optical properties of materials, researchers have achieved precise optical control through specialized structural designs, which results in high solar reflectivity and emissivity in the mid-infrared range. Aerogel structures possess exceptionally low solar transmittance (could be <0.01) while maintaining high mid-IR transmittance (>0.8), thereby enabling enhanced cooling performance under direct sunlight [8,9,10]. However, these aerogel-based systems must be expanded to millimeter-scale thickness, requiring specialized foaming or stretching processes that are more complex and costly than standard polymer film production. Furthermore, their application in large-area architectural or device-level systems faces additional mechanical challenges. PEA often possess very low mechanical strength—their structure resembles a porous gel or fragile “tofu-like” scaffold, which is highly susceptible to plastic deformation or collapse under compression or bending. This greatly limits their suitability for outdoor deployment or integration in construction-scale cooling systems, where mechanical robustness, dimensional stability, and repeatable processing are essential. Despite aerogels, photonic crystal and meta-surfaces also possess high optical efficacy, but the complex manufacturing process may limit large-scale, low-cost deployment [11,12,13]. Manufacturing porous structures using polymer materials presents a viable alternative. Porous structures achieve high solar reflection via light scattering mechanisms. Preparation techniques, including the template method and phase separation, are relatively simple but still require expensive equipment, posing challenges for large-scale production [14,15,16,17,18,19,20,21,22,23,24,25,26,27,28,29,30,31,32,33,34]. However, pure organic membranes are usually engineered for flexibility and wearability, offering water-wicking, and low hardness; these limitations severely hinder its feasibility as a durable top layer in building-integrated radiative cooling systems [33]. To improve its solar reflectivity and outdoor durability, inorganic pigments such as Al_2_O_3_, TiO_2_, BaSO_4_, CaCO_3_, and ZrO_2_ are considered as useful fillers [28,29,30,31,32]. Among them, TiO_2_ and Al_2_O_3_ are widely studied for their strong solar scattering capabilities and low cost [28]; however, their phonon absorption in the 8–13 µm infrared range makes them less favorable for infrared-transparent designs. In contrast, ZrO_2_ nanoparticles combine a high refractive index with low extinction coefficients in both the solar and mid-IR regions, making them attractive for dual-function coatings [29].

Despite these material options, most reported systems focus on either solar reflection or mid-IR emission, while few works have addressed the combination of IR transparency with scalable solar-reflective coatings, especially using low-cost, flexible fabrication methods such as spray-coating. This study aims to bridge that gap. Several recent works have begun to explore this integration. For instance, nanoporous polyethylene (P-PE) films have shown promising dual-function properties, offering high solar reflectance and mid-IR transmittance in cost-effective, flexible formats suitable for passive cooling applications [30]. TiO_2_/PDMS-based coatings have also demonstrated commercially scalable fabrication with solar reflectance exceeding 91% and IR emissivity over 75%, achieving sub-ambient cooling of 4–9 °C under direct sunlight [31,32]. Building on these developments, our work proposes a bilayer architecture combining a mid-IR–emissive PTFE substrate with a spray-coated zirconia/POE upper layer that enables solar reflection and IR transmittance simultaneously, while remaining compatible with scalable, low-cost fabrication strategies.

Moreover, the introduction of metamaterials offers a novel approach to radiation cooling. Metamaterial films fabricated through photolithography, nanoimprinting, or electron beam lithography demonstrate outstanding cooling performance [12,35,36,37,38,39,40,41,42]; however, their complex manufacturing processes and high costs remain significant barriers to widespread adoption. Thus, simplifying the preparation process and reducing equipment costs while maintaining cooling efficiency is a critical challenge for the large-scale implementation of radiation cooling technology [43,44].

To address this challenge, we propose a straightforward and scalable preparation process that enables cost-effective large-scale manufacturing. A composite membrane is fabricated by spraying a mixture of modified nano-zirconia and ethylene-octene copolymer (POE) dissolved in petroleum ether onto a polyethylene (PE) bubble film substrate. A cooling structure was prepared by covering the composite membrane onto a polytetrafluoroethylene (PTFE) plate which serves as an emitter. This structure exhibits a cooling capacity of 66.2 ± 4.3 W/m^2^. On-site testing demonstrated a temperature reduction of 3 ± 0.3 °C at noon compared to ambient conditions, with an average daily cooling effect of 4.7 ± 0.3 °C. This work advances the development of cost-effective and scalable radiation cooling technology, offering promising prospects for applications in building cooling and energy efficiency.

## 2. Methods and Materials

### 2.1. Principle Design

The radiative cooling structure proposed in this study consists of two functional layers: a top composite membrane composed of polyolefin elastomer (POE) and ZrO_2_ nanoparticles supported on a polyethylene (PE) bubble film, and a bottom polytetrafluoroethylene (PTFE) plate serving as the primary thermal emitter due to its high mid-infrared emissivity. While PTFE alone exhibits excellent spectral characteristics—namely, strong solar reflectivity in the 0.25–2.5 µm range and high emissivity in the atmospheric window of 8–13 µm—it remains ineffective in real outdoor environments due to its poor thermal insulation and strong susceptibility to convective heat gain from the ambient air. As a result, significant temperature reduction cannot be achieved using bare PTFE films under direct solar exposure.

To address this limitation, the upper composite membrane is designed to function as an infrared-transparent and solar-reflective cover that not only permits mid-infrared radiation emitted from PTFE to escape into the sky but also minimizes heat gain through solar absorption and convective transfer. The PE bubble substrate provides thermal insulation through its low thermal conductivity and internal air pockets, while the zirconia nanoparticles enhance solar reflectivity via Mie scattering. Furthermore, the inorganic nature of ZrO_2_ ensures excellent thermal stability and minimal infrared absorption across the 8–13 µm window, preventing the undesired re-radiation of heat back toward the PTFE layer.

Compared with common reflective pigments such as BaSO_4_, CaCO_3_, or Al_2_O_3_—which often exhibit phonon absorption bands in the infrared—ZrO_2_ offers a unique combination of high solar scattering efficiency and mid-infrared transparency. The selection of nanosized ZrO_2_ is based on Mie theory, where particles with diameters comparable to the solar wavelength range (0.4–2.5 µm) exhibit the highest scattering efficiency for solar spectrum range and transparency for mid-infrared range (8–13 µm). This ensures that the upper layer effectively reflects sunlight while allowing thermal radiation from the emitter to pass through, achieving passive radiative cooling without impeding mid-IR emissive performance.

While the conceptual use of PE foam as a substrate was introduced in early works [45], the main innovation of the present study lies in the development of a spray-based fabrication method for the reflective POE/ZrO_2_ layer. This method enables cost-effective, scalable manufacturing, offering significant advantages over conventional vacuum-based or templating techniques in terms of production feasibility and industrial applicability.

**Figure 1:**
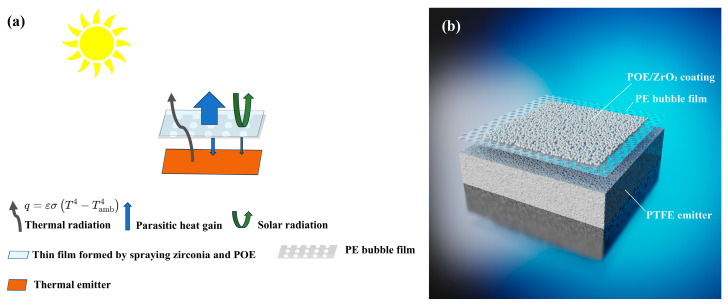
(**a**) Schematic diagram of the principal. (**b**) Schematic diagram of the composite film.

This study presents a daytime radiative cooling film based on a multi-layer functional composite structure, with its core principle realized through material synergy and optimized optical properties. The schematic diagram of the principle and film structure is shown in Figure 1. The radiative process illustrated in Figure 1 can be quantitatively described by the Stefan–Boltzmann equation

$$P=\varepsilon \sigma {\left(T-T_{\mathrm{amb}}\right)}^{4}$$

where *P* is radiative power emitted per unit area (W/m^2^), *ε* is emissivity of the surface (dimensionless, *σ* is Stefan–Boltzmann constant, *T* is absolute temperature of the radiating body (K), and *T*_amb_ is absolute temperature of the ambient space.

The underlying mechanism is detailed as follows:

1. Efficient Solar Reflection and Photothermal Suppression: A composite coating of polyolefin elastomer (POE) and nano-zirconia (particle size: 400 nm) is uniformly deposited onto the surface of polyethylene (PE) bubble film via spray-coating technology. Nano-zirconia, characterized by a high refractive index (n = 2.1) and an extremely low extinction coefficient in the UV–visible band (k ≈ 10^−4^–10^−6^), creates a robust scattering interface within the 200–2500 nm solar irradiation range (accounting for >95% of total solar energy). This significantly enhances the film’s reflectivity and effectively suppresses photothermal conversion. POE, serving as a flexible matrix, further improves light scattering efficiency by optimizing nanoparticle dispersion and simultaneously ensuring mechanical stability of the coating.
2. Collaborative Mid-Infrared Transmission and Thermal Radiation Dissipation: The composite membrane exhibits an average mid-infrared transmittance of 25% within the 8–13 μm atmospheric transparency window. The low extinction coefficient of nano-zirconia (k < 0.01) and the high transmittance of POE synergistically create an optical channel for efficient thermal radiation and heat dissipation from the underlying polytetrafluoroethylene (PTFE) emitter. PTFE exhibits ultra-high emissivity (ε ≥ 0.93) in this band, enabling the conversion of absorbed heat into mid-infrared photons that are directly radiated into low-temperature outer space (~3 K) through the atmospheric window, thereby achieving passive heat dissipation.
3. Thermal Conduction Blockage and Environmental Adaptability: PE bubble film acts as a supporting substrate, with its closed-cell structure imparting ultra-low thermal conductivity (κ ≈ 0.03 W/m·K). This effectively isolates thermal conduction interference from the external environment and maintains the directional radiation pathway for internal heat dissipation. Additionally, the lightweight and flexible nature of PE bubble film facilitates its application to complex surfaces, significantly enhancing its practical utility.
4. Collaborative Optimization Mechanism and Performance Advantages: This design achieves efficient daytime cooling through a triple synergistic effect of high reflection, high emission, and low thermal conductivity. The high reflectivity of the nano-zirconia and POE composite coating significantly minimizes photothermal absorption across the solar wavelength range. The synergistic mid-infrared transmittance of POE and zirconia ensures efficient radiative heat dissipation by PTFE. At the same time, the PTFE plate also has 72% reflectivity in the visible light band, which can reflect some sunlight that is not reflected by the surface coating again, further improving the overall reflectivity (Figure S1). The thermal insulation properties of PE bubble film block environmental thermal interference.

### 2.2. Preparation Process

#### 2.2.1. Modification of Nano Zirconia

Due to the hydrophilic nature of untreated zirconia particles and the hydrophobicity of the POE solution in petroleum ether, direct dispersion of ZrO_2_ led to severe agglomeration. To improve compatibility, zirconia was modified with stearic acid, introducing hydrophobic groups that enhance dispersion stability and promote uniform mixing within the polymer matrix. Stearic acid was selected as the surface modifier for zirconia nanoparticles due to its long-chain hydrophobic structure, which effectively adds -CH_2_ to the surface of ZrO_2_ and enhances compatibility with the POE solution in petroleum ether and facilitates uniform dispersion during spray-coating.

Disperse 10 g of unmodified 400 nm zirconia in 80 mL of ethanol and sonicate for 20 min. Simultaneously, dissolve 0.15 g of SA (stearic acid) (1.5% of the zirconia mass) in 20 mL of ethanol at 40 °C under continuous stirring. Once fully dissolved, combine the zirconia solution with the stearic acid solution and stir for 3 h. Subsequently, dry the mixture in an oven at 100 °C for further use. The purpose of surface modification of stearic acid on zirconia particles is to make zirconia hydrophobic, in order to enhance the suspension stability of the zirconia–POE (Ethylene-1-octene copolymer) mixture to be prepared in the next step.

#### 2.2.2. Spray Film

**Figure 2:**
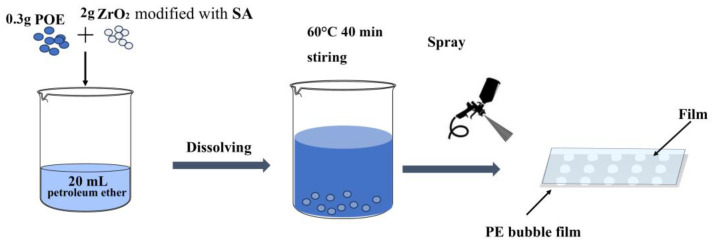
Schematic diagram of preparation process.

The preparation process is illustrated in Figure 2. Initially, 2 g of stearic acid-modified nano-zirconia and 0.3 g of POE (1.5% of the zirconia mass) are dispersed in 20 mL of petroleum ether solution. The mixture is heated to 60 °C and stirred for 40 min, followed by spray-coating onto the PE bubble film using a spray gun. After spray-coating, the PE bubble film is transferred to a well-ventilated area for drying, resulting in a composite membrane. This composite membrane will serve as a reflective layer for the cooling structure prepared in the next step.

POE was manufactured by Dow Co. (Shanghai, China). Petroleum ether (the boiling point: 60–90 °C) was manufactured by Macklin (Shanghai, China), PE bubble film was purchased from Shanghai Hengshitong Industry Group Co., Ltd. (Shanghai, China), ZrO_2_ powder was purchased from Ningbo Jinfeng New Material Technology Co., Ltd. (Ningbo, China), SA (stearic acid) was purchased from Sinopharm Group (Shanghai, China). PTFE Sheet was purchased from DuPont (Shanghai, China), commercial grade Teflon sheet.

### 2.3. Characterization and Measurements

Spectral characteristics, including solar reflectance (*R*_solar_) and long-wave infrared transmittance (TLWIR), were measured using a UV-Vis-NIR spectrophotometer (Lambda 750 S, PerkinElmer, Waltham, MA, USA) equipped with an integrating sphere and a Fourier transform infrared spectrometer (Thermo Scientific Nicolet iS50 FT-IR, Thermo Fisher Scientific Inc., Waltham, MA, USA). Surface morphology was analyzed using a scanning electron microscope (JSM-7500F (JEOL, Tokyo, Japan) and Zeiss Ultra Plus (Carl Zeiss AG, Oberkochen, Germany)), Contact Angle Goniometer (JY-82B Kruss DSA, Kruss GmbH, Hamburg, Germany). For powder samples, the IR spectral features were characterized via the KBr pellet method. Specifically, samples were ground into fine powders to ensure particle sizes below 2 μm for homogeneous mixing with spectroscopic-grade KBr (1–2 wt.% sample loading). The mixture was compressed into translucent pellets using a hydraulic press (10–20 MPa pressure for 2 min) to form uniform 13 mm diameter disks. Pellets were then analyzed on a Fourier transform infrared (FTIR) spectrometer with a resolution of 4 cm^−1^, collecting spectra from 4000 to 400 cm^−1^ with air as the background reference.

**Figure 3:**
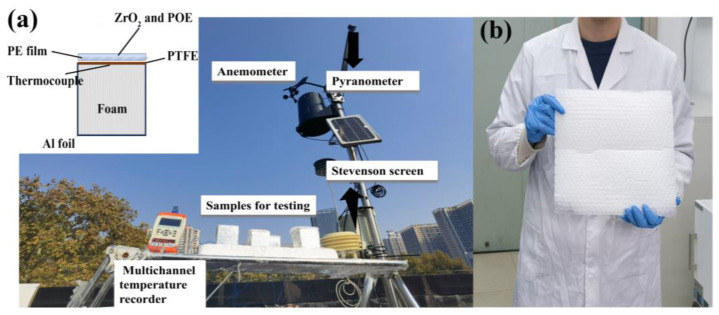
(**a**) Actual outdoor testing diagram and schematic diagram of testing equipment; (**b**) spray-coated samples.

The test sample was placed in an experimental box made of polystyrene foam plastic, with the outer surface covered with aluminum foil to reflect sunlight. The box was placed on a rack 1 m above the ground to isolate ground heat conduction and suppress nonradiative heat exchange effects on the test sample, such as sunlight absorption and air convection. A multichannel temperature tester (JK808, JINKO, Changzhou, China) and a K-type thermocouple (Omega Engineering, Inc., Shanghai, China) were used to monitor the temperature changes in the sample. The K-type thermocouple tester has a test range of −20~200 °C, with an error margin of ±0.3 °C. A louver box was used to record environmental temperature data, and a weather station was used to record weather data, such as solar radiation intensity, relative humidity, and wind speed. The outdoor experimental setup is shown in Figure 3.

## 3. Results and Discussion

The sub-ambient cooling performance observed in this work is attributed to the synergistic effect of three critical design principles: 1. High solar reflectance of the spray-coated POE/ZrO_2_ layer, which minimizes solar absorption and suppresses heating during daytime; 2. High mid-infrared emissivity of the underlying PTFE emitter, which facilitates effective thermal radiation into the sky through the atmospheric window; 3. Reduced convective heat gain, enabled by the use of a PE foam layer, which acts as a thermal insulator and diminishes parasitic heat exchange with ambient air, especially in open-air, no-insulation conditions.

To validate the three optical properties above and the effectiveness of the proposed radiative cooling structure, a series of experimental characterizations and performance evaluations were conducted. These include morphological and spectral analyses of the composite membrane, assessment of its hydrophobicity and structural stability, and thermal performance tests under both laboratory and outdoor conditions. Emphasis is placed on how the combination of the spray-coated reflective layer, the polyethylene (PE) foam substrate, and the polytetrafluoroethylene (PTFE) emitter contributes to the cooling performance. Through systematic analysis, the relationships between material structure, optical properties, and cooling effectiveness are elucidated, providing insights into the underlying mechanisms of the sub-ambient cooling effect.

### 3.1. Optical Property Modulation

**Figure 4:**
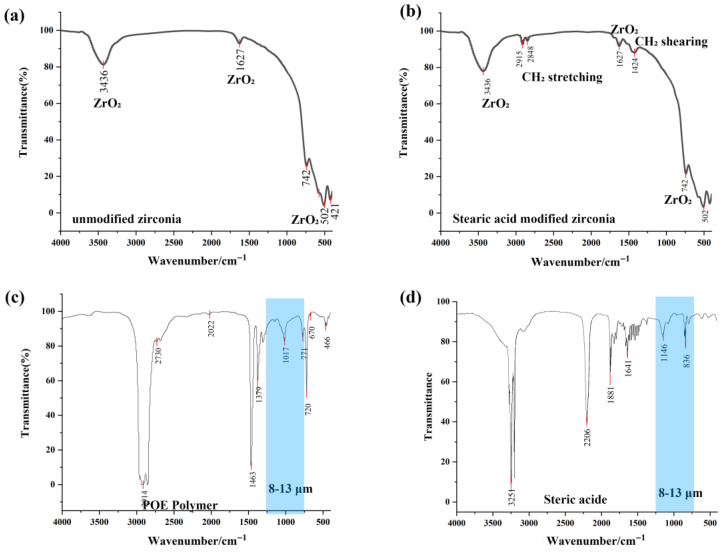
(**a**) Infrared absorption spectrum of unmodified zirconia; (**b**) absorption spectrum of zirconia modified with stearic acid; (**c**) POE infrared absorption spectrum; (**d**) infrared absorption spectrum of stearic acid. The blue areas in (**c**,**d**) show the mid-infrared wavelength range (8–13 μm).

A comparison of the infrared absorption spectra in Figure 4a,b reveals distinct absorption peaks for stearic acid-modified ZrO_2_ at 2915 cm^−1^, 2848 cm^−1^, and 1424 cm^−1^. The absorption peaks at 2915 cm^−1^ and 2848 cm^−1^ correspond to the asymmetric and symmetric stretching vibrations of the CH_2_ groups in stearic acid molecules, respectively. The absorption peak at 1424 cm^−1^ is attributed to the shearing vibration of the CH_2_ groups in stearic acid molecules. The presence of these characteristic absorption peaks confirms the successful modification of stearic acid on the ZrO_2_ surface, with its alkyl chain displaying typical vibrational features in infrared spectroscopy, thereby verifying the effective binding of stearic acid to ZrO_2_. Figure 4c displays the infrared spectrum of POE7447, showing minor absorption peaks at 771 cm^−1^, 1017 cm^−1^, and 1149 cm^−1^ within the 8–13 μm wavelength range (corresponding to wavenumbers of 769–1250 cm^−1^). Overall, POE7447 demonstrates high transparency across this spectral band. Figure 4d illustrates the infrared spectrum of SA (stearic acid), revealing minor absorption peaks at 797 cm^−1^, 837 cm^−1^, 854 cm^−1^, and 1146 cm^−1^, while maintaining high transparency across the spectrum. This high transparency is a key factor in selecting stearic acid as a surface modifier. The high infrared transparency of stearic acid allows for effective surface modification of ZrO_2_ without substantially altering its optical properties [46], thereby establishing a robust foundation for subsequent functional applications.

**Figure 5:**
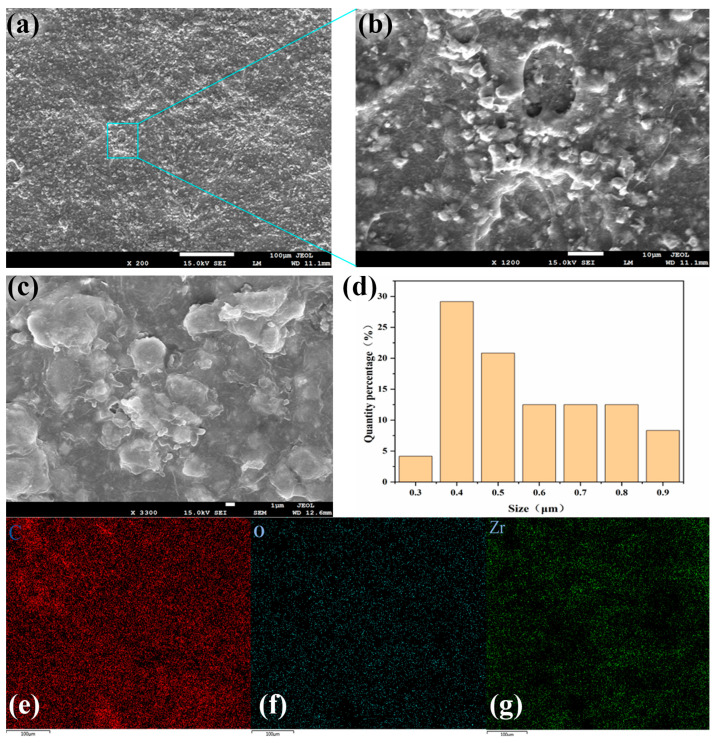
(**a**–**c**) Scanning electron microscope image of sprayed radiative cooling layer; (**d**) pore size distribution diagram of spray film; (**e**–**g**) EDS elemental mapping of C, O and Zr.

Figure 5 provides a systematic investigation of the morphological and compositional characteristics of the spray-coated composite material. In Figure 5a, the spray-coating process produces a highly rough surface with numerous pores, which significantly increases the number of air–material interfaces and enhances surface roughness, which promotes multiple scattering of incident sunlight [47]. Such surface features are beneficial for solar reflectance, as they reduce the chance of light penetration and increase the probability of reflection on the surface. Figure 5b illustrates that the high-velocity impact of sprayed particles forms localized craters on the polyolefin elastomer (POE) surface, while partial encapsulation of zirconia particles by POE leads to microscale protrusions. Figure 5c reveals a heterogeneous distribution of zirconia particles of varying sizes on the POE surface, along with localized agglomeration into larger clusters. Figure 5d highlights that non-agglomerated zirconia particles dominate the surface, whereas agglomerated regions show a significant reduction in scattering efficiency. This phenomenon stems from the spray-coating process, where the slurry impacts the polyethylene (PE) bubble film and spreads out, thereby causing the initially dispersed zirconia particles to cluster. Such agglomeration adversely affects the material’s overall reflectivity. It should be emphasized that industrial-scale implementation will mitigate this challenge through process parameter optimization, including reduced nozzle pressure (to minimize droplet splashing) and the addition of rheological modifiers to improve slurry homogeneity. These strategies are well-documented in ceramic thin-film spraying processes [48,49]. Figure 5e,f further examines the spatial distribution of zirconium (Zr) elements through elemental mapping. Although zirconia is generally uniformly distributed across the surface, localized deficiencies are observed in certain regions. These deficiencies spatially correlate with agglomerated zirconia clusters, where dense particle packing hinders uniform dispersion, resulting in compositional inhomogeneity.

**Figure 6:**
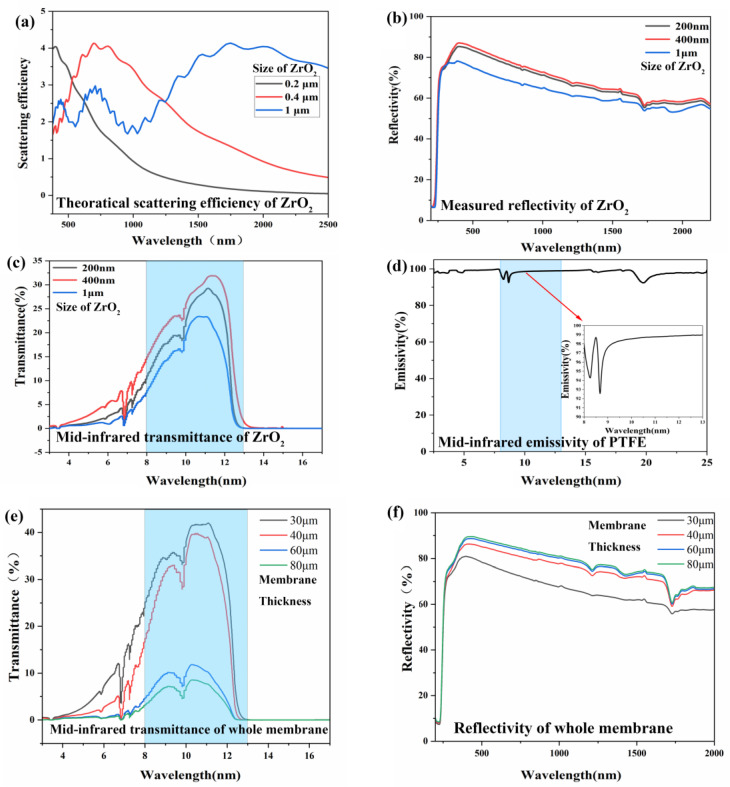
(**a**) The theoretical scattering efficiency of ZrO_2_ nanoparticles with varying sizes within the POE matrix was calculated; (**b**) the reflectivity of ZrO_2_ nanoparticles with varying sizes during the spray-coating process. (**c**) The mid-infrared transmittance of ZrO_2_ nanoparticles with varying sizes at a thickness of 40 μm. (**d**) The mid-infrared emissivity of PTFE in its role as the emitter. (**e**) The mid-infrared transmittance of whole membrane at different thicknesses. (**f**) Reflectivity of whole membrane at different thicknesses. The blue areas in (**c**,**d**) show the mid-infrared wavelength range (8–13 μm).

Figure 6 systematically investigates the light scattering characteristics and radiative cooling performance of zirconia nanoparticles (ZrO_2_ NPs) with different particle sizes within a polyolefin elastomer (POE) matrix. Theoretical calculations (Figure 6a) demonstrate that the scattering efficiency of ZrO_2_ NPs exhibits a redshift phenomenon with increasing particle size, achieving optimal solar radiation scattering capability at 400 nm where the scattering cross-section significantly increases while absorption loss minimizes. Experimental validation (Figure 6b) reveals that 400 nm ZrO_2_ NPs attain an average reflectance of 82.3% in the UV–Vis spectrum, outperforming systems containing 200 nm (80.7%) and 1 μm (72.5%) particles. Notably, although 200 nm NPs underwent stearic acid surface modification, their high specific surface area still induces pronounced agglomeration effects, resulting in practical scattering performance comparable to the 400 nm system. Conversely, 1 μm NPs maintain superior dispersion stability due to reduced surface energy, with experimental results showing excellent agreement with theoretical predictions. The definition of average reflectance, transmittance, and emissivity can be found in Note S1.

Infrared spectral analysis (Figure 6c) demonstrates that under 40 μm film thickness, the 400 nm system achieves an average transmittance of 23.0% in the 8–13 μm atmospheric window, surpassing 200 nm (19.0%) and 1 μm (14.9%) systems. This enhancement originates from the synergistic interaction between optimized Mie scattering effects and phonon resonance absorption. Further thickness-dependent studies (Figure 6e,f) reveal that increasing coating thickness from 30 μm to 80 μm elevates UV–vis reflectance from 76.0% to 86.9%, while 8–13 μm transmittance decreases from 30.7% to 5.2%. These optical responses arise from multiple physical mechanisms: (1) Enhanced photon scattering probability in thicker films amplifies surface reflectance; (2) Cumulative Fresnel reflections at ZrO_2_ (~2.1–2.2)/POE (~1.5) interfaces intensify with increasing layer numbers due to significant refractive index contrast; (3) Coordinated phonon resonance absorption (ZrO_2_) and vibrational absorption (C-H/C-O bonds in POE) prolong infrared photon absorption paths, drastically reducing transmittance. For comparison, Figure 6d shows PTFE substrate exhibits exceptional 94.2% emissivity in the 8–13 μm band, providing critical reference for constructing high-efficiency radiative cooling systems.

### 3.2. Outdoor Cooling Test

**Figure 7:**
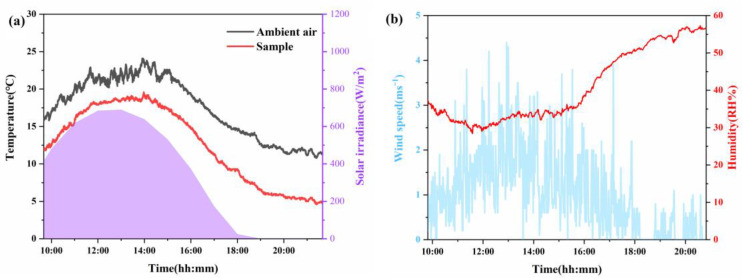
(**a**) Temperature curves of outdoor cooling test, with solar irradiance showing as purple areas. (**b**) Wind speed and humidity curve during the outdoor test.

To assess the operational efficacy of the thermal management system under real-world conditions, an 11 h outdoor field evaluation was performed on the rooftop of Wuhan University of Technology (30.52° N, 114.34° E), commencing at 09:40 local time on 29 November 2024. As schematically depicted in Figure 3, the experimental configuration minimized non-radiative heat transfer through three principal design elements: (i) elevation of the sample on a 1 m platform, (ii) encapsulation within a reflective aluminum foil-clad foam substrate, and (iii) full enclosure using a spray-coated polyethylene (PE) bubble film, while a polytetrafluoroethylene (PTFE) radiative emitter was vertically integrated atop the assembly. Throughout the evaluation period, precision thermocouples were utilized for continuous temperature monitoring of the PTFE emitter, complemented by ambient temperature and relative humidity measurements acquired via a Stevenson screen-equipped meteorological station. Concurrent solar irradiance and wind speed profiles were obtained at 1 min intervals using a Class A pyranometer and cup anemometer, respectively, integrated into the onsite meteorological monitoring system (Figure 3a). Quantitative analysis (Figure 7a,b) revealed that during peak insolation events (689 W/m^2^; 33% RH), the system sustained a mean sub-ambient temperature differential of 3.6 °C, culminating in a diurnal average cooling performance of 4.7 ± 0.3 °C relative to ambient conditions. Based on the measured irradiance (600 W/m^2^) and atmosphere transmission τ = 0.8, the calculated net cooling power was 66.2 ± 4.3 W/m^2^. These findings substantiate the symbiotic functionality between the spray-deposited PE cushion reflective coating and PTFE-based radiative architecture, which collectively facilitated sustained ambient thermal regulation across diurnal cycles. Furthermore, Figure 3b showcases a scalable 30 × 27 cm^2^ prototype manufactured via spray-coating, exhibiting robust interfacial adhesion and dimensional uniformity—critical metrics validating the technology’s compatibility with industrial-scale radiative cooling material fabrication. In order to avoid the chance of the test results, Supplementary Data for November 27 (Figure S3) is provided, which shows a typical afternoon decline in solar intensity (1:50–5:00 p.m.) and stable radiative cooling performance. Both data sets confirmed that the material achieved effective cooling during the day, even in non-ideal weather.

To bridge the laboratory characterization and outdoor test performance, we correlated the optical properties of the composite membrane with its observed cooling effect. As measured in the laboratory, the membrane exhibits a high solar reflectance (average 82.3%) and a mid-infrared transmittance of approximately 23% in the 8–13 µm atmospheric window. These parameters, when integrated into a theoretical radiative cooling model (as detailed in Note S2), yield a net cooling power of 66.2 ± 4.3 W/m^2^ under standard atmospheric conditions.

This theoretical cooling power was substantiated by field experiments, in which the membrane–emitter structure achieved a consistent sub-ambient temperature difference of 3.6 °C at peak solar irradiance and an average daily cooling effect of 4.7 ± 0.3 °C. The consistency between the predicted and observed performance validates the effectiveness of the optical design and highlights the practical potential of the proposed material system.

### 3.3. Additional Performance

**Figure 8:**
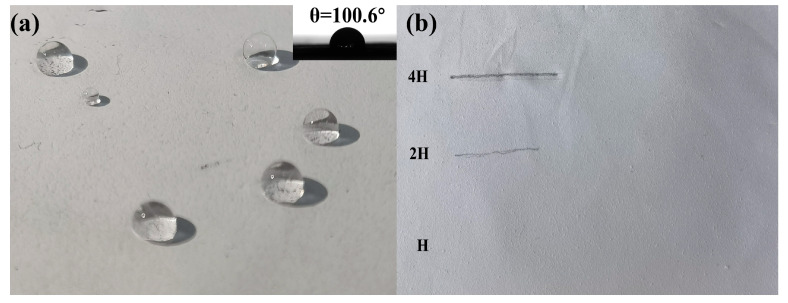
(**a**) Water contact angle; (**b**) pencil test hardness chart.

As shown in Figure 8a, contact angle measurements reveal a surface contact angle of 100.6°, demonstrating pronounced hydrophobicity that indicates low water wettability of the material surface. This characteristic effectively mitigates water residue adhesion and corrosion risks in outdoor environments, fulfilling operational requirements for exterior applications. Further mechanical evaluation via the pencil hardness test (Figure 8b) demonstrates distinct scratch formation under 4H pencil loading, indicating a hardness below the 4H grade. Mild scratches generated by 2H pencils confirm an intermediate hardness between 2H and 4H. Notably, no observable surface damage occurs under H pencil testing, verifying a minimum hardness of H grade. These results collectively demonstrate that the coating achieves a balanced mechanical profile: it maintains sufficient hardness (≥H) to resist daily abrasion while avoiding excessive rigidity (>4H) that could induce brittle fracture during assembly or maintenance. Its mechanical properties are also supplemented in the Supplementary Materials (Figure S4). This optimized hardness–toughness equilibrium ensures both structural integrity and process compatibility in practical applications.

In addition to the demonstrated hydrophobicity (contact angle of 100.6°) and mechanical robustness (pencil hardness ≥ H), the structural and material properties of the composite film suggest promising long-term durability for outdoor applications. The hydrophobic surface minimizes moisture adsorption and contamination, which are critical for maintaining optical and thermal performance under real-world conditions.

Moreover, the composite structure, featuring flexible POE and thermally stable nano-zirconia particles, provides resistance to cracking and mechanical fatigue. While long-term outdoor exposure and aging tests were beyond the scope of this study, the preliminary results indicate that the membrane possesses essential attributes for sustained field use. Future work will include accelerated weathering tests and extended outdoor deployments to further assess environmental resilience and practical longevity.

For the probability of UV damage, the upper layer consists of POE and ZrO_2_ nanoparticles on a polyethylene (PE) bubble film. While ZrO_2_ itself is a UV-stable inorganic oxide known for its chemical and photothermal durability, POE and PE are both polymeric materials that may undergo photodegradation under prolonged UV exposure. The polymer backbone can be cleaved, and chromophoric groups may be formed, potentially increasing solar absorptivity and reducing transparency in the mid-infrared range. Notably, the lower PTFE layer is chemically inert and well-known for its excellent long-term UV and thermal stability, as evidenced by its widespread use in outdoor photovoltaic and aerospace applications. The main concern of UV damage lies in the upper protective/transmissive layer.

For installation on outdoor surfaces, the upper layer is applied directly onto the PTFE emitter via spray-coating, forming a self-supporting flexible film. The coating demonstrates sufficient adhesion to PTFE due to the elastomeric nature of POE, without requiring additional adhesives. For fixed installations (e.g., on roofs or containers), the bilayer film can be directly laid over the target surface and optionally fixed with adhesives, mechanical clips, or tension frames depending on the application scale.

### 3.4. Spray-Coating Cost

Through comprehensive cost–benefit analysis, this work establishes the economic feasibility of scalable radiative cooler fabrication via an optimized spray-coating methodology. The manufacturing protocol, schematically illustrated in Figure 2, involves three sequential, optimized steps: (i) surface functionalization of zirconia (ZrO_2_) nanoparticles through stearic acid modification (0.15 wt.%) in ethanol solution (10 mL/g ZrO_2_); (ii) formulation of sprayable ink by dispersing functionalized ZrO_2_ with polyolefin elastomer (POE, 0.15 g/g ZrO_2_) in petroleum ether (10 mL/g ZrO_2_); and (iii) controlled deposition onto polyethylene (PE) bubble cushion substrates using air-assisted spray-coating (0.6–0.8 MPa) with integrated polytetrafluoroethylene (PTFE) radiative emitter layers.

To estimate the actual chemical reagent consumption per unit area, we considered the densities and mixing ratio of the two primary components: zirconia (ZrO_2_) nanoparticles and ethylene-octene copolymer (POE). According to the formulation, POE was used at a mass ratio of 0.15 g per gram of ZrO_2_. The densities of ZrO_2_ and POE were assumed to be 5.85 g/cm^3^ and 0.86 g/cm^3^, respectively. This corresponds to a ZrO_2_ content of 2.896 g/cm^3^ and a POE content of 0.434 g/cm^3^ in the final solid membrane. Therefore, the reagent consumption per square meter of coated area is estimated to be the following:

ZrO_2_: 40 cm^3^ × 2.896 g/cm^3^ = 115.8 g

POE: 40 cm^3^ × 0.434 g/cm^3^ = 17.4 g

Cost analysis based on optimized parameters—including 5 min spray cycles, 100 cm^2^ deposition area per cycle, ZrO_2_ consumption of 2 g/cycle, 40 μm coating thickness, and equipment durability (1000 operational hours for spray system)—reveals a total manufacturing cost of $77.56/m^2^. This cost structure comprises material costs ($75.73/m^2^) and equipment depreciation ($1.83/m^2^). Material cost breakdown analysis can be seen in Table 2 and Table 3. This cost-optimized profile represents a significant advancement over conventional radiative cooling materials (typically exceeding $200/m^2^; please refer to Table 4 for specific comparison). This calculation is for the laboratory preparation process cost. Compared with other methods, large-scale manufacturing can be achieved using only a spray gun and an air compressor (as shown in Figure S5), demonstrating obvious equipment cost advantages.

In laboratory, ethanol and petroleum ether as solvents were used only once without any recycling. However, in industrial-scale production, solvents such as ethanol and petroleum ether can be effectively recycled using distillation or solvent recovery systems. A recent review demonstrates that solvent recovery rates of 80–90% for ethanol and high efficiency for other volatile organics are routinely achievable [52]. (Additionally, directly purchasing modified zirconia instead of modifying it in-house can further reduce costs.) The price for bulk purchase of zirconia is $16.8/kg [45]. The cost after considering solvent recovery is approximately $26/m^2^. When coupled with the demonstrated scalability of spray-coating—validated through 810 cm^2^ (30 cm × 27 cm) functional prototypes—this cost-effective manufacturing approach provides critical technological and economic foundations for large-scale deployment of radiative cooling systems in urban heat mitigation and energy-efficient building applications.

## 4. Conclusions

This study proposes a cost-effective and scalable fabrication method for radiative cooling membranes via spray-coating technology. The observed sub-ambient cooling effect results from a rationally engineered structure that simultaneously enhances solar reflectance, enables efficient mid-IR radiation, and suppresses convective heat exchange. The integration of a solar-reflective yet IR-transparent spray-coated layer with a PE foam substrate and PTFE emitter provides a cost-effective and scalable solution, suitable for real-world passive cooling applications without requiring additional insulation enclosures. In this work, the radiative cooling structure was fabricated by spray-coating a mixture of modified nano-zirconia and ethylene-octene copolymer (POE) dissolved in petroleum ether onto a polyethylene (PE) bubble film substrate, with polytetrafluoroethylene (PTFE) serving as the emitter. This structure exhibits a cooling power of 66.2 ± 4.3 W/m^2^. On-site testing demonstrated a midday temperature reduction of 3 °C compared to ambient conditions, with an average daily cooling effect of 4.7 ± 0.3 °C. The estimated production cost of $77.56 per square meter is significantly lower than alternative fabrication approaches. The industrial manufacturing cost is expected to be approximately $26/m^2^, showing good potential for large-scale production. The material costs in this study exhibited a less pronounced reduction compared to other research endeavors. However, it demonstrates significant economic advantages in equipment expenditure (with remarkable cost-effectiveness relative to alternative methodologies that often require equipment costing tens of thousands of dollars). Concurrently, the simplified fabrication process and shorter processing time collectively present a novel fabrication strategy for large-scale implementation of radiative cooling films. This approach particularly highlights the synergistic optimization between economic viability and manufacturing efficiency in thin-film production technology.

## Figures and Tables

**Table 1:** Comparison of representative radiative cooling materials.

Material Type	Example Materials/Structures	Optical Characteristics	Fabrication Methods	Remarks
Inorganic pigments	Al_2_O_3_, TiO_2_, BaSO_4_, CaCO_3_, and ZrO_2_	High solar reflectance	Ball milling and dispersion, co-precipitation and calcination	Low-cost; high thermal stability; need backing material
Organic materials	Porous PDMS, P-PVDF, and PE aerogel	Medium or high solar reflectance, high mid-IR transmission, flexible, and weather-resistant; can serve as freestanding films	Phase separation, fiber stretching, spray-coating	Suitable for flexible applications; direct coating without backing
Hybrid (organic–inorganic)	Organic matrix + inorganic nanoparticles (e.g., ZrO_2_)	Hight solar reflectance and IR Emission	Composite spraying, rolling	Enhanced performance through structure optimization

**Table 2:** Prices of various raw materials and equipment (data from references [45,50,51]).

Material	Price	Manufacturer and Purity
POE	$3.1/kg	Dow Chemical, Engage 8200, Shanghai, China
PE bubble film	$0.095/m^2^	Shanghai Hengshitong (no purity marker), Shanghai, China
ZrO_2_ powder	$37.5/kg	Ningbo Jinfeng, ≥99.9% purity, Ningbo, China
ethanol	$0.009/mL	Sinopharm, ≥99.7% purity, Shanghai, China
stearic acid	$16.8/kg	Macklin, ≥98%purity, Shanghai, China
petroleum ether	$0.052/mL	Macklin, boiling point: 60–90 °C, Shanghai, China
PTFE sheet	$0.58/m^2^	DuPont (China), Teflon, Shanghai, China
W71 spray gun	$93.5/each	/
air compressor	$126.4/each	/

**Table 3:** Usage of various raw materials and equipment.

Material	Usage per m^2^	Price per m^2^	Notes
POE	17.4 g	$0.05394	0.15 g/g ZrO_2_
PE bubble film	1 m^2^	$0.095	
ZrO_2_ powder	115.8 g	$4.3425	
ethanol	1158 mL	$10.422	10 mL/g ZrO_2_
stearic acid	1.09 g	$0.018312	0.15 wt.%
petroleum ether	1158 mL	$60.216	0.63–0.66 g/cm^3^ 10 mL/g ZrO_2_
PTFE sheet	1 m^2^	$0.58	
W71 spray gun and air compressor	/	$1.83	5 min spray cycles, 100 cm^2^ deposition area per cycle
sum	$77.56 per m^2^		

**Table 4:** Prices of other reported cooling membrane (data from reference [50]).

Material	Price
BaSO4-acrylic resin (5 cm × 5 cm × 400 μm)	$210.91/m^2^
CaCO3-acrylic resin (5 cm × 5 cm × 400 μm)	$146.35/m^2^
PDMS sponge (d = 5.4 mm, h = mm)	$171.00/m^2^
PDMS-aluminum foil	$362.47/m^2^
P-PVDF (8 cm × 8 cm × 700 μm)	$153.84/m^2^
Spray POE&ZrO2-PE bubble pad-TFE	$132.1/m^2^

## Data Availability

The original contributions presented in this study are included in the article/Supplementary Material. Further inquiries can be directed to the corresponding authors.

## Supplementary Materials

The following supporting information can be downloaded at: https://www.mdpi.com/article/10.3390/ma18184385/s1, Note S1: The definition of average reflectance, transmittance, and emissivity; Note S2: The theoretical calculation of net cooling power; Note S3: Estimation of Uncertainty in Cooling Power and Temperature Measurements; Figure S1: The reflectivity of PTFE, the average reflectance of sunlight band is 72%; Figure S2: The infrared transmittance of the sprayed bubble pad is 75.8% on average in the 8–13 μm wavelength range; reflectance of PE bubble pad in sunlight band; Figure S3: Outdoor cooling diagram on the roof of an experimental building in Wuhan Institute of Technology, 27 November 2024; Figure S4: Stress–strain curve (Tensile strength 0.32 MPa, elongation at break > 150%); Figure S5: Air compressor and spray gun.

## Abbreviations

POE	ethylene-octene copolymer
PE	polyethylene
PTFE	polytetrafluoroethylene
PDMS	polydimethylsiloxane
UV	ultraviolet
SA	stearic acid
NP	nanoparticle
P-PVDF	porous polyvinylidene fluoride
