# Supplementary material for: Low-Cost Scalable Radiative Cooling Membrane via Spray Fabrication for Sustainable Thermal Management
Source: Materials (Basel). 2025 Sep 19;18(18):4385. doi: 10.3390/ma18184385 (PMC12472148; doi:10.3390/ma18184385)
Supplement: Supplementary file 1 [file materials-18-04385-s001.zip › materials-3830493-supplementary.pdf]

Supplementary Materials

Low-Cost Scalable Radiative Cooling Membrane via Spray Fabrication for Sustainable Thermal Management

NoteS1. The definition of average reflectance, transmittance and emissivity:

The average transmittance ($\bar{T}$) over the 8–13 μm atmospheric infrared window is calculated from spectral transmittance data via integral or discrete summation methods, as follows:

$$\bar{T} = \frac{1}{\lambda_2 - \lambda_1} \int_{\lambda_1}^{\lambda_2} T(\lambda) d\lambda \qquad \text{(S1)}$$

where:

- λ_1=8 μm, λ_2=13 μm
- T(λ) is the spectral transmittance at wavelength λ.

The average solar reflectance($\bar{R}_{solar}$),denoted as:

$$\bar{R}_{solar} = \frac{\int_{0.3\mu m}^{2.5\mu m} I_{solar}(\lambda) R_{solar}(\lambda) d\lambda}{\int_{0.3\mu m}^{2.5\mu m} I_{solar}(\lambda) d\lambda} \qquad \text{(S2)}$$

In Eq. (S2), λ represents the solar light band from 0.3-2.5 μm, $I_{\mathrm{solar}}(\lambda)$ is the normalized ASTM G173 global solar intensity spectrum, and $R_{\mathrm{solar}}(\lambda)$ is the spectral reflectance of the device.

The average emissivity in the longwave infrared atmospheric transmission window, $\bar{\varepsilon}_{LWIR}$ is defined as:

$$\bar{\varepsilon}_{LWIR} = \frac{\int_{8\mu m}^{13\mu m} I_{BB}(T,\lambda) \varepsilon_{LWIR}(\lambda) d\lambda}{\int_{8\mu m}^{13\mu m} I_{BB}(T,\lambda) d\lambda} \qquad \text{(S3)}$$

In Eq. (S3), $I_{\mathrm{BB}}(T,\lambda) = \frac{2hc^2}{\lambda^5} \frac{1}{e^{hc/\lambda k_B T} - 1}$, is the spectral radiation intensity of a black body at temperature T (assumed to be 25°C) as defined by Planck's Law.h is Planck's constant, k_B is Boltzmann's constant, c is the speed of light in a vacuum, and $\varepsilon_{LWIR}(\lambda)$ is the emissivity of the device in the atmospheric window range of 8-13 μm.

The emissivity of the material, $\varepsilon(\lambda)$, is given by Kirchoff's law of thermal radiation:

$$\varepsilon(\lambda) = \alpha(\lambda) = 1 - \tau(\lambda) - R(\lambda) \qquad \text{(S4)}$$

In Eq. (S4), $\alpha(\lambda)$ is the absorption rate, $\tau(\lambda)$ is the transmission rate, $R(\lambda)$ is the reflectance rate.

Note S2. The theoretical calculation of net cooling power:

The net cooling power (***P_{net}***) of a radiative cooler can be calculated by the equation below:

$$Q_{net} = Q_r - Q_a - Q_{nonrad} - Q_{sun} \qquad \text{(S5)}$$

Q_{sun} is the solar power absorbed by the sample (including Spray coating and PE bubble pad and PTFE). Q_{sun} =0(at night), $Q_{sun} = P_{sun} \cdot \varepsilon_1$ (daytime), P_{sun}=450W/m^2, ε_1 is the solar absorption coefficient. Q_{sun} is given in the form of an exponential distribution.

Q_{nonrad} is nonradiative heating power obtained by the sample from the surrounding media.

$$Q_{nonrad} = h(T_{amb} - T_r) \qquad \text{(S6)}$$

where h is the non-radiant heat transfer coefficient (($\boldsymbol{h} = \frac{\mathbf{1}}{\boldsymbol{R_1}+\boldsymbol{R_2}+\frac{\mathbf{1}}{\boldsymbol{Qconv}}}$). Due to the thin coated ZrO_2&POE film, only the thermal resistance of the bubble film is considered ($R_1 = n * \frac{0.002}{0.04}$). n is the number of bubble film layers. A layer of bubble film is 0.002 m thick and the thermal conductivity is 0.04 W/m/K. There is an air layer of 500μm in between λ_{air} = 0.023W/m/K, $R_2 = \frac{5\times10^{-6}}{0.023}$), Q_{nonrad} is the surface heat transfer coefficient.

Q_a is the incident atmospheric radiation absorbed by the sample. Atmospheric window transparency is set to 0.8.

$$Q_\alpha = 2\Pi \int_0^{\frac{\pi}{2}} sin\theta cos\theta d\theta \int_0^{\infty} B(T_\alpha,\lambda)e_r(\lambda,\theta)e_\alpha(\lambda,\theta)d\lambda. \qquad \text{(S7)}$$

Q_r is the radiative power emitted by the sample.

$$Q_r = P_{r\mathrm{ZrO2\&poe}} + \varepsilon_2 P_{r\,\mathrm{bubble\ film}} + \varepsilon_2\varepsilon_3^n P_{r\mathrm{PTFE}} \qquad \text{(S8)}$$

Considering the IR masking of the upper layer to the lower material, $\varepsilon_2$is the IR transmittance of the ZrO_2 &POE film and ε_3 is the IR transmittance of the bubble film (0.76).

Note S3: Estimation of Uncertainty in Cooling Power and Temperature Measurements

To evaluate the experimental accuracy and reproducibility of the reported cooling performance, we performed uncertainty analysis on both the temperature reduction and the calculated net cooling power.

1. Temperature Measurement Accuracy

The temperature reduction values were obtained from thermocouples (JK808 multi-channel temperature tester), with a manufacturer-specified measurement accuracy of **±0.3 °C**. The **sub-ambient temperature difference of 3.6 °C** represents a peak value observed under maximum solar irradiance and is not subject to statistical averaging. Therefore, no error margin is provided for this threshold value.

In contrast, the **average daily cooling effect of 4.7 °C** was determined over an 11-hour measurement period. Based on the thermocouple accuracy and stable ambient conditions, the corresponding uncertainty is **±0.3 °C**.

2. Cooling Power Estimation and Error Range

The theoretical cooling power P_c was calculated using the following relationship:

$$P_c = Ah_c(T_{amb} - T)$$

Where:

Two test cases were modeled:

- November 27, irradiance = 400 W/m² → cooling power = **54.2 W/m²**
- November 29, irradiance = 600 W/m² → cooling power = **66.2 W/m²**

Taking **66.2 W/m²** as the representative value corresponding to the November 29 test (the primary field validation), and accounting for temperature sensor uncertainty **±0.3 °C**, we estimate the cooling power uncertainty to be **±4.3 W/m²**. The dominant contributor to this uncertainty is temperature uncertainty.

FigureS1. Optical properties of PTFE

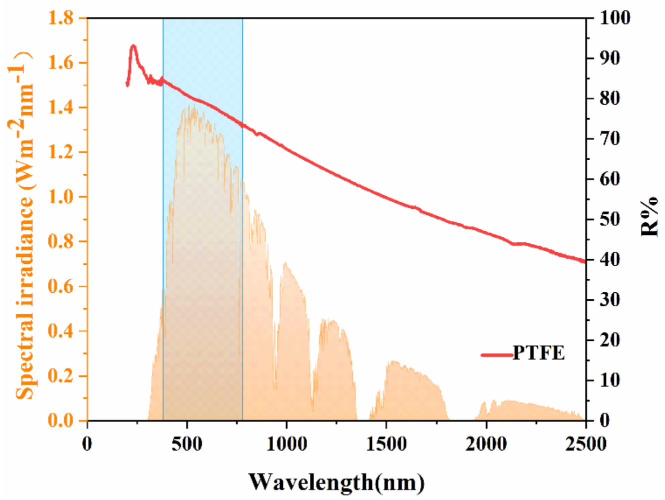

Fig S1 The reflectivity of PTFE, the average reflectance of sunlight band is 72%.

FigureS2. Infrared transmittance of PE bubble film

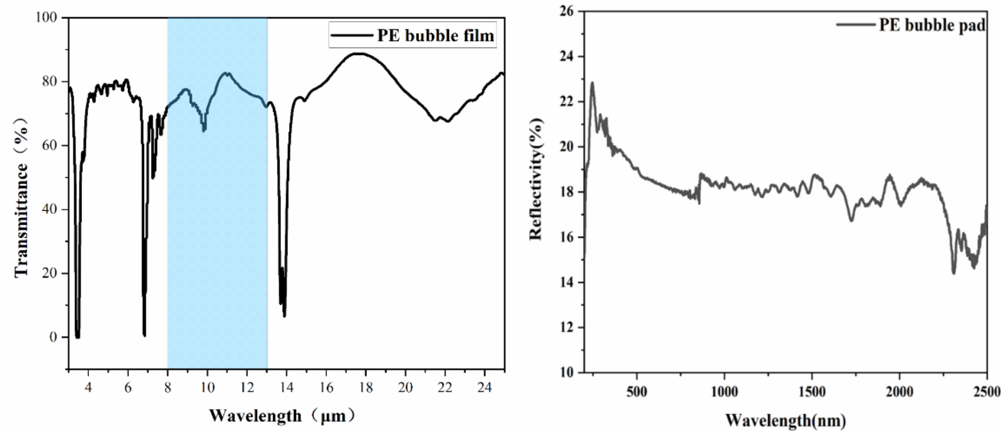

Fig S2 The infrared transmittance of the sprayed bubble pad is 75.8% on average in the 8-13 μ m wavelength range; Reflectance of PE bubble pad in sunlight band.

FigureS3. 11.27 Outdoor test chart

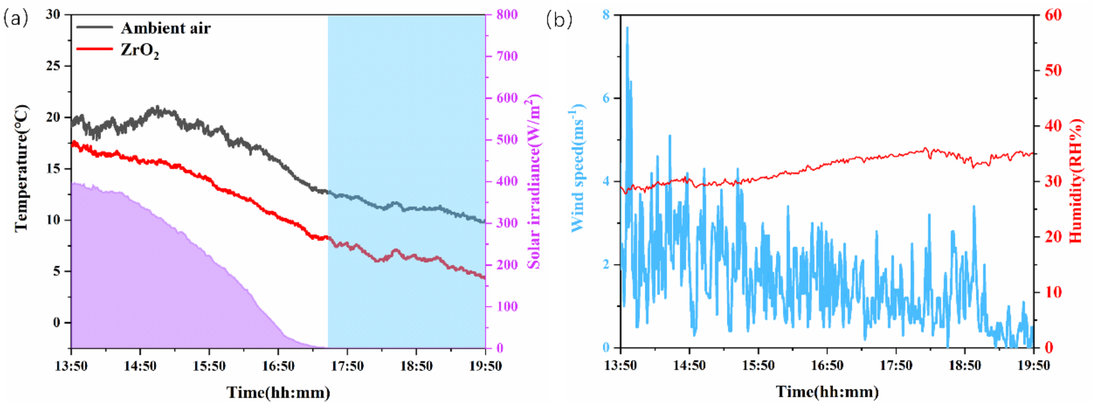

Fig S3 Outdoor cooling diagram on the roof of an experimental building in Wuhan Institute of Technology, Nov 27, 2024

FigureS4. Mechanical Properties

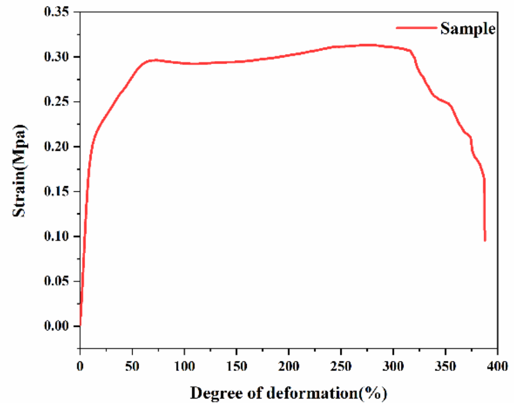

Fig S4 Stress-strain curve (Tensile strength 0.32MPa, elongation at break >150%)

FigureS5. Photo of spraying tool

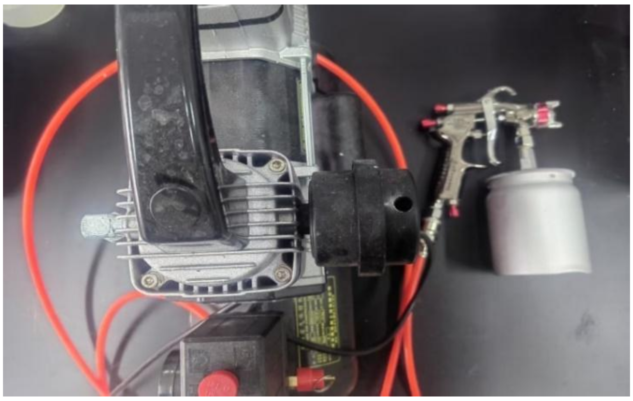

Fig S5 Air compressor and spray gun
